# Successful Removal of an Ovarian Tumor

**Published:** 1867-09

**Authors:** 


### SUCCESSFUL REMOVAL OF AN OVARIAN TUMOR.

Dr. Seely read a report of a case of ovarian tumor, which had been successfully removed by Dr. Burnham.

The patient, 31 years of age, had married at the age of 20, and borne four children. In September, 1864, she suffered from the presence of an abdominal tumor. As it developed it became necessary to perform the operation of paracentesis repeatedly, to relieve the great tension, nausea, vomiting, and vaginal prolapsus, caused by the excessive ascites. For a year or more she was in this way rendered quite comfortable. During the last five months, however, the operation had been performed sixteen times, removing at each time fourteen quarts of serous fluid. The tumor was removed on the 30th of May last. An incision of the usual length having been made through the walls of the abdomen, the tumor was carefully withdrawn, the peduncle ligated, and then divided with the knife. The wound in the abdomen was closed by means of silver sultures, support being given by means of a bandage around the body. After the operation there was scarcely any prostration. Patient slept well; pulse continued a few days somewhat more frequent than normal. There was also considerable intestinal pain, which was readily relieved. The recovery was perfect, in four weeks.

